# LINC01977 Promotes Breast Cancer Progression and Chemoresistance to Doxorubicin by Targeting miR-212-3p/GOLM1 Axis

**DOI:** 10.3389/fonc.2021.657094

**Published:** 2021-03-31

**Authors:** Zheng Li, Yaming Li, Xiaolong Wang, Yiran Liang, Dan Luo, Dianwen Han, Chen Li, Tong Chen, Hanwen Zhang, Ying Liu, Zekun Wang, Bing Chen, Lijuan Wang, Wenjing Zhao, Qifeng Yang

**Affiliations:** ^1^ Department of Breast Surgery, General Surgery, Qilu Hospital of Shandong University, Jinan, China; ^2^ Pathology Tissue Bank, Qilu Hospital of Shandong University, Jinan, China; ^3^ Research Institute of Breast Cancer, Shandong University, Jinan, China

**Keywords:** long non-coding RNAs, breast cancer, bioinformatics, chemoresistance, miR-212-3p, GOLM1

## Abstract

Long non-coding RNAs(lncRNAs) play an important role in cancer initiation and progression. However, hub lncRNAs involved in breast cancer still remain underexplored. In this study, integrated bioinformatics analysis was used to define LINC01977 as a key oncogenic driver in breast cancer. Subsequently, *in vitro* assays showed that LINC01977 could significantly promote breast cancer progression and chemoresistance to doxorubicin. To further investigate its biological mechanism, we performed dual-luciferase reporter assay, real-time PCR, RNA immunoprecipitation (RIP), and rescue assay. Our results indicated that LINC01977 may function as ceRNA to prevent GOLM1 gene from miRNA-mediated repression by sponging miR-212-3p. Overall, LINC01977 can serve as a novel prognostic indicator, and help develop more effective therapeutic approaches for breast cancer patients.

## Introduction

Breast cancer was reported as the leading cause of cancer-related death among women worldwide (1). Although conventional cancer therapies including surgery, radiotherapy and chemotherapy have vastly improved, many patients with breast cancer still have poor clinical outcomes (2–4). Consequently, we should continue to investigate the mechanisms of breast cancer malignant progression to develop more effective therapeutic strategies for breast cancer patients.

Long non-coding RNAs (lncRNAs) are classified as a subtype of non-protein coding RNAs longer than 200 nucleotides in length, which have been found to regulate various biological processes including immunity, apoptosis, autophagy, and cell proliferation (5–7). Moreover, emerging evidence showed that lncRNAs dysregulation contributed to cancer malignant progression by regulating gene expression at epigenetic, transcriptional, and post-transcriptional levels (8–10). For instance, Sun Z et al. reported that lncRNA MALAT1 could promote angiogenesis and epithelial–mesenchymal transition *via* regulating YAP1-MALAT1-miR-126-5p axis in colorectal cancer (11). Xiao G et al. revealed that lncRNA TTTY15 upregulation was associated with the malignant progression of prostate cancer by sponging the microRNA let-7 to suppress miRNA-mediate CDK6 and FN1 degradation (12). Wang J et al. demonstrated that H19 lncRNA(H19), a highly abundant and conserved imprinted gene, contributed to tamoxifen resistance in breast cancer patients *via* inducing autophagy activation (7). Additionally, Tsai KW et al. identified that LINC00659 promoted cell proliferation and suppress cell apoptosis by activating PI3K-AKT signaling pathway in colon cancer (13).

MicroRNAs(miRNA) are small (19–25 nucleotides) non-protein coding RNAs that commonly affect the process of gene expression *via* gene translation silencing or mRNA degradation (14, 15). Since their discovery, miRNA have been proved to involve in many pathological processes, such as carcinogenesis (16). To our knowledge, altered miRNA expression have been found in most tumor types, including breast cancer. Mechanically, miRNA can load onto the RNA-induced silencing complex (RISC) where they are directly associated with the Argonaute (AGO) family proteins (17). Once they capture a target mRNA, the RISC Argonaute RNase may initiate the process of mRNA degradation. Intriguingly, lncRNAs are able to inhibit miRNA-mediate mRNA degradation *via* binding to miRNA in RISC, namely competing endogenous RNAs (ceRNAs) mechanism (18). Thereby, it is essential to further explore their interactive mechanism for yielding a promising therapeutic avenue for breast cancer patients.

Doxorubicin (DOX), an anthracyclic antitumor antibiotic, is one mainstream chemotherapeutic drug, either in single or combined regimen (19). DOX kills cancer cells by causing DNA damage that typically lead to cell cycle arrest or cell death (20). During tumor evolution, some tumor cells would escape from DNA damage response network to acquire chemoresistance (21). Previous studies reported that the drug transporter ABCB1 can export DOX from cells and then contribute to resistance to DOX (22). However, ABCB1 inhibitor failed to reverse unresponsiveness to DOX in cancer treatment. Currently, accumulating evidence indicated that the aberrant expression of lncRNAs was associated with the development of drug resistance (23), which further exploration can provide a novel means to reduce tumor recurrence after treatment with DOX.

In this study, we performed integrated bioinformatics analysis to select LINC01977, which was significantly upregulated in breast cancer samples and DOX resistant cell lines. Subsequently, the biological function of LINC01977 was evaluated by *in vitro* experiments. Finally, our findings indicated that LINC01977 could promote breast cancer progression and chemoresistance to DOX by targeting miR-212-3p/GOLM1 axis. Overall, LINC01977 may act as a novel prognostic indictor and potential therapeutic target for breast cancer patients.

## Methods

### Gene Expression Dataset and Data Processing

Breast cancer RNA-seq count data (level3) and corresponding clinical information were obtained from The Cancer Genome Atlas (TCGA, http://cancergenome.nih.gov/). These data were normalized by edgeR package (24) and then transformed by voom function in limma package (25). Different analysis for 112 tumors and paired normal samples was performed using limma algorithm (25). LncRNAs with FDR <0.05 and absolute log2(fold changes) >1 were considered as significant difference. TCGA pancancer and GTEx datasets were downloaded from UCSC Xena (http://xena.ucsc.edu/). GSE155478 microarray data containing three DOX resistant cell lines and three DOX sensitive cell lines were gained from the GENE EXPRESSION OMNIBUS (GEO, https://www.ncbi.nlm.nih.gov/geo/). Here, we re-annotated probe sets of GPL22755 by mapping all probes to the human genome (GRCh38) with SeqMap method (26). All probes were mapped to the genome without mismatch, and the probes mapped to protein-coding and pseudogene transcripts were removed. The drug response data were sourced from the Genomics of Drug Sensitivity in Cancer (GDSC, https://www.cancerrxgene.org/).

### Cell Lines and Cell Culture

In this study, all cell lines were obtained from American Type Culture Collection (ATCC). MDA-MB-231 and MCF-7 cells were cultured in Dulbecco’s modified Eagle’s medium (DMEM) (Invitrogen, USA) containing 10% fetal bovine serum (Hyclone), 100 U/ml penicillin, and 100 μg/ml streptomycin. MCF-10A, MCF-10AT, MCF-10CA1A, and MCF-10CA1H cell lines were cultured in DMEM/F12 (Invitrogen, Carlsbad, California) containing 5% horse serum (Invitrogen), 500 ng/ml hydrocortisone (Sigma-Aldrich, St. Louis, Missouri), 100 ng/ml cholera toxin (Sigma-Aldrich), 10μg/ml insulin (Invitrogen), and 20 ng/ml epidermal growth factor (EGF, Sigma-Aldrich). All cells used for the experiments were cultured in a humidified incubator with 5% CO2 at 37°C.

### Plasmid Establishment and Transfection

All plasmids were sourced from Vigene Biosciences (Rockville, Maryland). The LINC01977 sequence was cloned into PCDH vector, and then transfected into MDA‐MB‐231 or MCF‐7 using Lipofectamine 2000 (Invitrogen, MA, USA). Empty PCDH vector was used as negative control. PLKO.1 lentiviral plasmids for control or carrying shRNA sequences for LINC01977 were used to infect MDA‐MB‐231 or MCF‐7 cells according to the provided protocol. The short hairpin RNAs (shRNA) sequence targeted to LINC01977 are listed as follows:

Sh-1, 5’-TGTTCCTAATTTGGACACTGGTTTA‐3’;
Sh-2, 5’-AATGAGAAACCAGATACCATGGAAT‐3’

### Cell Proliferation and Cytotoxicity Assay

3-(4,5-dimethylthiazol-2-yl)-2,5-diphenyltetrazolium (MTT) assay was used to assess cell viability. In 96-well plates, the transfected cells were plated at a density of 2×10^3^ cells per well and then incubated overnight. Subsequently, the medium was replaced by the solutions containing indicated concentrations of DOX. After incubation for the indicated time, 20μl MTT (5mg/ml) was added into each well and incubated for another 4-6h. The supernatants were aspirated and 100 μl of dimethyl sulfoxide (DMSO) was add into each wells. Microplate Reader (Bio-Rad, Hercules, CA, USA) was used to measure the absorbance values at 490nm.

### Colony Formation Assay

The transfected cells were seeded in 6-well plates at a density of 800 cells/well and incubated for two weeks. Then they were washed with PBS, fixed with methanol, and stained with 0.1% crystal violet. After excess staining was washed with PBS, images were obtained with a microscope.

### Ethynyl Deoxyuridine Incorporation Assay

For cell proliferation analysis, the EDU incorporation assay was performed using an EDU assay kit (Ribobio, Guangzhou, China) following the manufacturer’s protocols. Breast cancer cells were seeded in 96-well plates at a density of 1×10^4^ cells/well. After incubation in DMEM with EDU labeling for 2h, the cells were fixed, permeated, and stained with Apollo Dye Solution. Lastly, the nucleic acid was labeled with Hoechst33342. Laser scanning microscope was applied to observe the treated cells.

### Apoptosis Assay

Cell apoptosis assay was performed using Annexin V Apoptosis Detection Kit (BD Biosciences, NJ, USA) according to the manufacturer’s instruction. Briefly, the transfected cells were stained with 5μL Annexin V-FITC and 5μL PI, followed by collecting and washing with ice cold PBS. Then cells were incubated in the dark for 15 min. Lastly, apoptosis cells were measured *via* flow cytometry. Further data analysis was evaluated using FlowJo 7.6.1 software.

### Transwell Assay

*In vitro* migration ability was assessed using a migration assay, which was performed using transwell inserts (8-μm pore size, Corning Costar, USA) in 24-well plates (Corning Costar, USA). 1×10^5^ MDA-MB-231 or 2×10^5^ MCF-7 were suspended in 200μL serum-free medium and seeded into the inside of each insert, while 700μL medium containing 20% FBS was placed in the lower well. After incubation for 24-72h, the infiltrating cells, on the lower surface, were fixed with methanol and stained with 0.1% crystal violet. Cell invasion assay was conducted using the same procedure as in the cell migration assay, except that the inside of each insert was coated with Matrigel. After the infiltrating cells were photographed, ImageJ software was used to count the number of cells.

### Wound Healing Assay

For the wound-healing assay, transfected cells were seeded in 24-well plates and cultured in full DMEM medium supplemented with 10% FBS until a confluent monolayer was achieved. Then cells were scratched with a sterile 10 µl sterile micropipette tip, washed with PBS, and replenished with serum-free DMEM. The images were captured by phase-contrast microscope at both 0 and 24h and the wound area was measured by the Image J software.

### RNA Immunoprecipitation Assay

For RNA immunoprecipitation (RIP) assay, the procedure was carried out using the Magna RIP RNA‐Binding Protein Immunoprecipitation Kit (Millipore, Billerica, Massachusetts) following the manufacturer’ protocol. Antibodies against Ago2 and IgG were purchased from Millipore. The coprecipitated RNAs (total RNA) was extracted for the detection of miRNA and lncRNA expressions by real-time PCR.

### Dual-Luciferase Reporter Assay

Full length LINC01977 sequence with wild‐type miR-212-3p binding sites were synthesized and fused to the luciferase reporter vector pmirGLO (Promega, Madison, Wisconsin). Wild‐type LINC01977 constructs and miR‐212‐3p mimic cotransfected into breast cancer cells. After incubation for 24h, transfected cells were plated in 96-well plates. According to the manufacturer’s instructions, the dual‐luciferase reporter assay system (Promega) was used to measure the firefly and Renilla luciferase activities at 48h after transfection. Renilla luciferase activity was employed to normalize against firefly luciferase activity.

### RNA Extraction, Reverse Transcription, and Real-time PCR Analysis

Total RNA from breast cancer cells was extracted using The TRIzol reagent (Invitrogen). Complementary DNA (cDNA) of lncRNA and mRNA was reversely transcribed using PrimeScript reverse transcriptase (RT) reagent kit (TaKaRa, Shiga, Japan), while cDNA of miRNA was reversely transcribed using the Prime‐Script miRNA cDNA Synthesis Kit (TaKaRa). Biosystems StepOne plus System was employed to perform real-time PCR. Primers used for real-time PCR are listed in **Supplementary Table 1**.

### Protein Isolation and Western Blot

Western blot assay was conducted as previously described (27). Briefly, equivalent amounts of total cellular protein from each sample were separated on 10% SDS-PAGE gels and transferred onto a PVDF membrane (Millipore). After 5% non-fat milk was used to block non-specific binding sites, membranes were incubated with specific primary antibodies overnight at 4°C and appropriate secondary antibodies for 2 hours at room temperature. The protein bands were detected using enhanced chemiluminescence (ECL; Bio-Rad, USA). The antibodies used in the experiments are available in **Supplementary Table 2**.

### WGCNA Network Construction and Module Preservation Analysis

Here, DESeq2 (28) and preprocessCore packages were used to normalize RNA-seq count data from TCGA. Genes with zero variance between high- and low- groups were removed, and the first 25% genes with median absolute deviation (MAD) value at least greater than 0.01 were retained. Subsequently, WGCNA was performed according to the previous description (6). Briefly, two weighted gene co-expression networks were established according to GOLM1’s median expression value, the low-expression group serving as the reference network and the high-expression group serving as the test network. After defining 5 as an optimum soft threshold power, a scale-free network and topological overlap matrix (TOM) were constructed. In this study, we identified 12 modules in the group with high GOLM1 expression and 13 modules in the group with low GOLM1 expression by hierarchical clustering analysis (**Supplementary Figures1D, E** **)**. Module preservation analysis in WGCNA package was used to evaluate the preservation of gene pairs between two networks. In order to make more accurate result, permutation testing with 1000 times was performed. Z-summary score was applied to evaluate which modules were the lowly preserved module. Z-summary values less than 10 indicated that this module had weak preservation. Finally, clusterprofiler package (29) was employed to perform the gene ontology(GO) analysis for top100 eigengene-based connectivity (kME) genes associated with the lowly preserved module.

#### Statistical Analysis

All the experiments in the study were repeated at least thrice. Experiment data were presented as the mean ± SD (standard deviation). Continuous data between two groups were assessed using Student’s t-test and multiple groups comparison were performed using Kruskal-Wallis test. Comparison between survival curves was carried out using the Kaplan–Meier method followed by the log-rank test. Statistical analysis was performed by GraphPad Prism 8 and R software (Version 3.6.1). Differences with p value < 0.05 were considered statistically significant.

## Results

### LINC01977 Functioned as an Oncogenic Driver and Predicted a Poor Prognosis in Breast Cancer Patients

**Figure 1:**
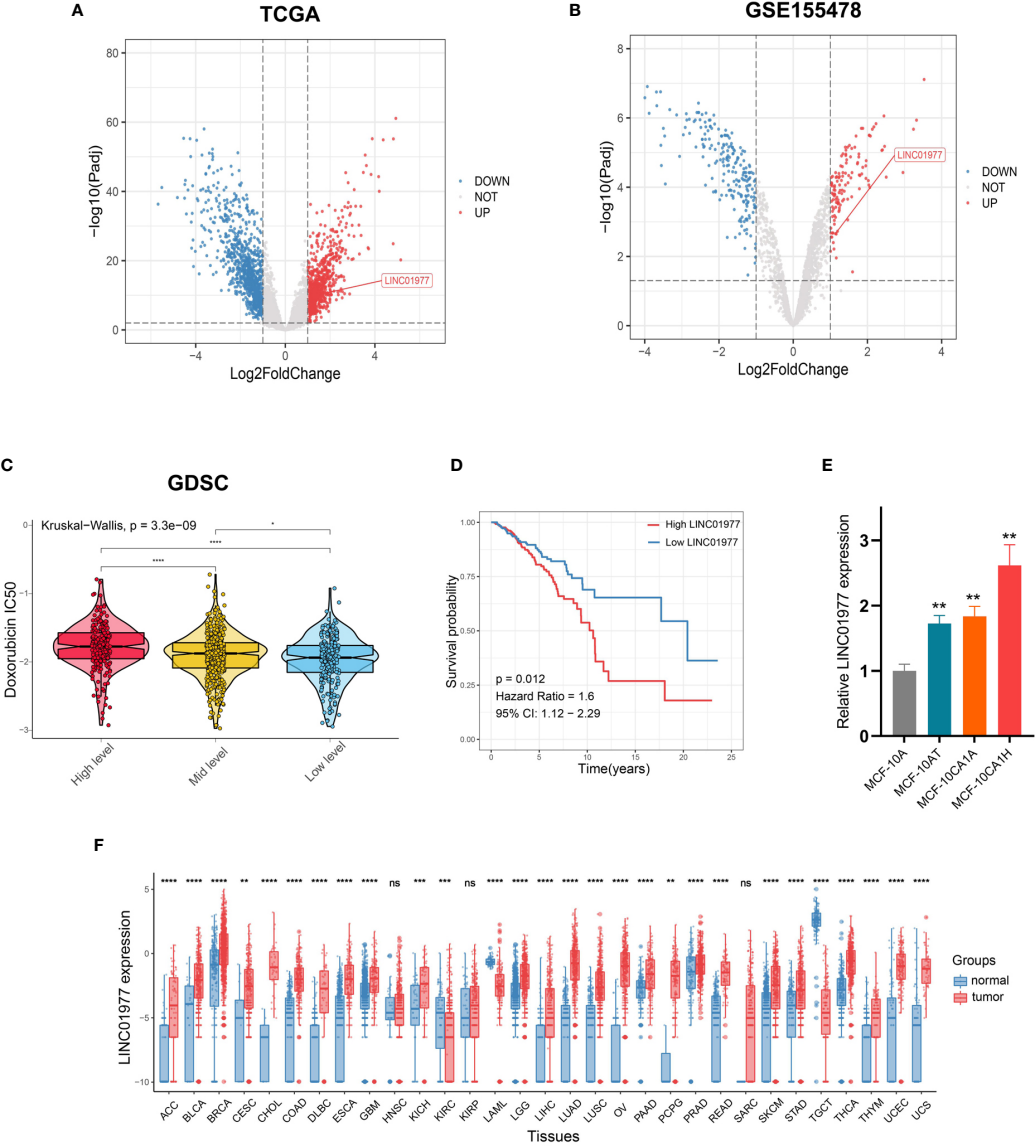
LINC01977 functioned as an oncogenic driver and predicted a poor prognosis in breast cancer patients. **(A)** Different analysis between 112 tumors and paired normal samples from TCGA breast cancer data. **(B)** Different analysis between DOX sensitive and resistant cell lines from GSE155478. **(C)** IC50 value for DOX of TCGA breast cancer patients was estimated on the basis of GDSC drug response data. Three subgroups represented the expression level of LINC01977. **(D)** Kaplan-Meier survival analysis in TCGA breast cancer patients. **(E)** Expression profiles of LINC01977 in breast cancer progression cell line model. **(F)** Expression profiles of LINC01977 in pancancer dataset. (*p < 0.05, **p < 0.01, ***p < 0.001, ****p < 0.0001; Student’s t-test).

We performed different analysis based on limma package in TCGA and GSE155478 datasets, respectively (**Figures 1A, B** **;** **Supplementary Figure 1A**). Of upregulated genes, 18 lncRNAs were overlapped (**Supplementary Figure 1B**). Our results indicated that LINC01977 expression was significantly upregulated in breast cancer samples and DOX resistant cell lines (**Figures 1A, B** **)**. LINC01977 is located at 17q25.3 in humans consisting of three exons with 1799bp in length (**Supplementary Figure 1C**). Based on the GDSC drug response data, the high expression group of LINC01977 represented a higher IC50 value for DOX than other subgroups (**Figure 1C**). Kaplan-Meier survival analysis showed that patients with high LINC01977 expression had shorter survival time than those with low LINC01977 expression (**Figure 1D**). Subsequently, breast cancer progression cell line model was enrolled into this study. Real-time PCR result suggested that LINC01977 upregulation may play an important role in the onset and progression of breast cancer (**Figure 1E**). The Pancancer Analysis of transcriptional level revealed that LINC01977 expression was commonly upregulated in most cancer samples compared to their normal samples, including breast cancer (**Figure 1F**).

### LINC01977 Knockdown Reduced Breast Cancer Cell Proliferation, Metastasis, and Chemoresistance to DOX

**Figure 2:**
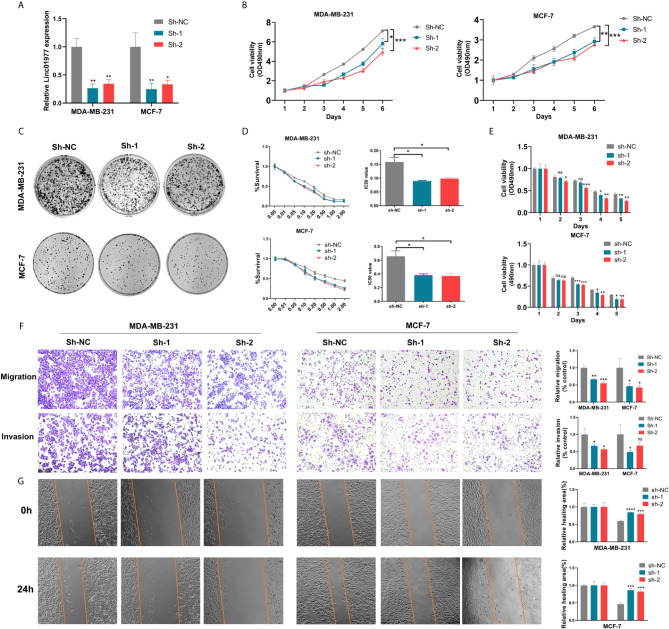
LINC01977 knockdown reduced breast cancer cell proliferation, metastasis, and chemoresistance to DOX. **(A)** The efficiency of shRNA knockdown was evaluated by real-time PCR in MDA-MB-231 and MCF-7 cells. **(B)** MTT assay was used to evaluate cell viability of MDA-MB-231 and MCF-7 transfected by control or knockdown plasmids. **(C)** Colony formation assay displayed the influence of LINC01977 knockdown on breast cancer cell proliferation. **(D)** IC50 value for DOX in MDA-MB-231 and MCF-7 transfected by control or knockdown plasmids. **(E)** Cytotoxicity assay was used to test the suppression of DOX resistance caused by LINC01977 knockdown in MDA-MB-231 and MCF-7 cells. DOX concentration: 0.1μM in MDA-MB-231 and 0.5μM in MCF-7 cells. **(F)** Cell migration and invasion assays. **(G)** Wound healing assay. (*p < 0.05, **p < 0.01, ***p < 0.001, ****p < 0.0001; Student’s t-test).

To further identify the biological role of LINC01977 in breast cancer, MDA-MB-231 and MCF-7 cell lines were selected and stably transfected with control or knockdown vectors. The efficiency of shRNA knockdown was evaluated by real-time PCR (**Figure 2A**). MTT and colony formation assays demonstrated that LINC01977 knockdown resulted in a decreased cell growth rate and less colony formation numbers (**Figures 2B, C** **)**. The cytotoxicity assay showed that LINC01977 downregulation made breast cancer cells more sensitive to DOX and represented a decreased IC50 value compared to the control group (**Figures 2D, E** **)**. Besides, decreased metastasis capability was observed in the LINC01977 knockdown group *via* transwell and wound healing assays (**Figures 2F, G** **)**. Taken together, our results revealed that LINC01977 knockdown can inhibit cell proliferation, metastasis, and resistance to DOX in breast cancer cells.

### LINC01977 Overexpression Promoted Breast Cancer Cell Proliferation, Metastasis, and Chemoresistance to DOX

**Figure 3:**
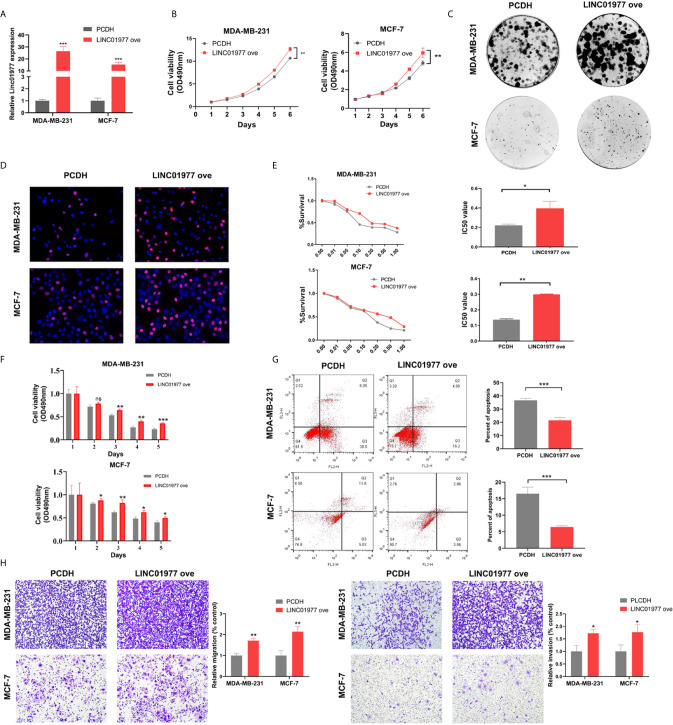
LINC01977 overexpression promoted breast cancer cell proliferation, metastasis, and chemoresistance to DOX. **(A)** The efficiency of LINC01977 expression was evaluated by real-time PCR in MDA-MB-231 and MCF-7 cells. **(B)** MTT assay. **(C)** Colony formation assay. **(D)** EDU assay. **(E)** IC50. **(F)** Cytotoxicity assay. **(G)** Apoptosis assays. As shown in right panel, total apoptosis cells were quantified. **(H)** Transwell assay. Left panel: Cell migration assay. Right panel: Cell invasion assay. PCDH: empty PCDH vector using as negative control. (*p < 0.05, **p < 0.01, ***p < 0.001, ****p < 0.0001; Student’s t-test).

Here, we transfected MDA-MB-231 and MCF-7 cell lines with control or overexpression vectors. The efficacy of LINC01977 overexpression was confirmed by real-time PCR in two cell lines (**Figure 3A**). MTT, colony formation, and EDU assays revealed that LINC01977 had a significant positive effect on cell proliferation of MDA‐MB‐231 and MCF‐7 cells (**Figures 3B–D** **)**. Accordant with the knockdown results, LINC01977 overexpression increased IC50 value for DOX in MDA‐MB‐231 and MCF‐7 cells (**Figure 3E**). Meanwhile, the group with LINC01977 overexpression displayed a stronger resistance to DOX than the control group in a time dependent manner (**Figure 3F**). Furthermore, LINC01977 impeded DOX-induced cell apoptosis, which was confirmed by flow cytometry (**Figure 3G**). In the transwell assay, we observed increased tumor cell migration and invasion in the group with LINC01977 overexpression (**Figure 3H**). To sum up, our findings demonstrated that LINC01977 upregulation could promote cell proliferation, metastasis, and resistance to DOX in breast cancer cells.

### LINC01977 Served as a Molecular Sponge *via* Binding to miR-212-3p

**Figure 4:**
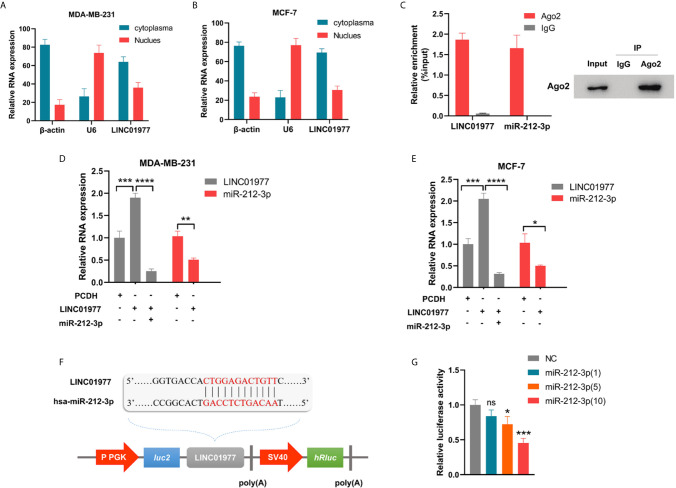
LINC01977 served as a molecular sponge *via* binding to miR-212-3p. **(A, B)** The subcellular location of LINC01977. β-actin: positive control for cytoplasm; U6: positive control for nuclear. **(C)** Left panel: Real-time PCR analysis of LINC01977 and miR-212-3p enriched by Ago2 proteins in MCF-7 cells. Right panel: Western blot was used to confirm the specific immunoprecipitation of Ago2. **(D, E)** Real-time PCR confirmed the interaction between LINC01977 and miR-212-3p in MDA-MB-231 and MCF-7 cells. **(F)** A schematic of wild-type LINC01977 luciferase reporter vectors. **(G)** Dual-luciferase reporter assay. NC: negative control of miR-212-3p mimics. (*p < 0.05, **p < 0.01, ***p < 0.001, and ****p < 0.0001; Student’s t-test).

Cellular fractionation assay revealed that LINC01977 was mainly localized in the cytoplasm rather than nucleus (**Figures 4A, B** **)**. This result suggested that LINC01977 may exert its downstream function by post-transcriptional regulation. RegRNA2.0 speculated that LINC01977 had potential binding site with the seed sequence of miR-212-3p. RNA-binding protein immunoprecipitation (RIP) assay was performed to investigate whether LINC01977 and miR-212-3p existed in the RNA‐induced silencing complex (RISC), which main component was Argonaute2 (AGO2) (30). As presented in **Figure 4C**, the anti-AGO2 group pulled down more amounts of LINC01977 and miR-212-3p than the IgG group, indicating that LINC01977 might sponge miR‐212‐3p existed in RISC to inhibit downstream mRNA degradation. Subsequent real-time PCR confirmed that LINC01977 overexpression was reversed by transfection of miR‐212‐3p mimics, whereas miR-212-3p expression was repressed by transfection of LINC01977 overexpression vectors (**Figures 4D, E** **)**. To further investigate the relationship between LINC01977 and miR-212-3p, the luciferase reporter plasmids containing their putative binding site were constructed (**Figure 4F**). Dual luciferase reporter assay showed that luciferase activity was evidently decreased with the raising amount of transfected miR‐212-3p mimics (**Figure 4G**).

### LINC01977 Rescued GOLM1 Expression *via* ceRNA Mechanism

**Figure 5:**
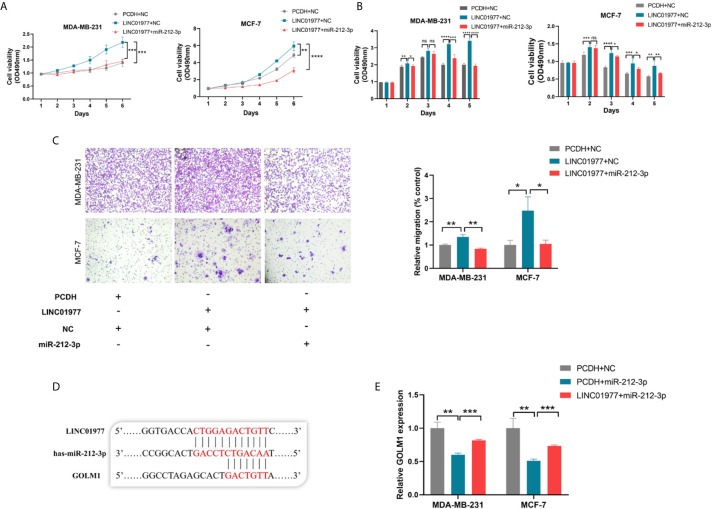
LINC01977 rescued GOLM1 expression *via* ceRNA mechanism. **(A)** Promotion of cell proliferation by LINC01977 was rescued by miR-212-3p expression. **(B)** Promotion of DOX resistance by LINC01977 was rescued by miR-212-3p expression. **(C)** Promotion of cell migration by LINC01977 was rescued by miR-212-3p expression. **(D)** The predicted binding site for GOLM1. **(E)** Real-time PCR was used to identify that GOLM1 expression could be suppressed by miR-212-3p expression and promoted by LINC01977 expression. (*p < 0.05, **p < 0.01, ***p < 0.001, ****p < 0.0001; Student’s t-test).

To identify the biological function of miR‐212‐3p, miR-212-3p mimics was transfected into MDA‐MB‐231 and MCF‐7 cells. As shown in **Figures 5A-C**, we found that miR-212-3p was able to attenuate LINC01977-induced cell proliferation, migration, and chemoresistance to DOX in MDA‐MB‐231 and MCF‐7 cells. TargetScanHuman 7.2 was used to conjecture that miR-212-3p could combine with the 3’ untranslated region (UTR) of GOLM1 (**Figure 5D**). Subsequently, real-time PCR showed that miR-212-3p suppressed GOLM1 expression, whereas this effect can be partly rescued by LINC01977 expression (**Figure 5E**).

### LINC01977 Promoted Breast Cancer Progression and Chemoresistance to DOX by Targeting miR-212-3P/GOLM1 Axis

**Figure 6:**
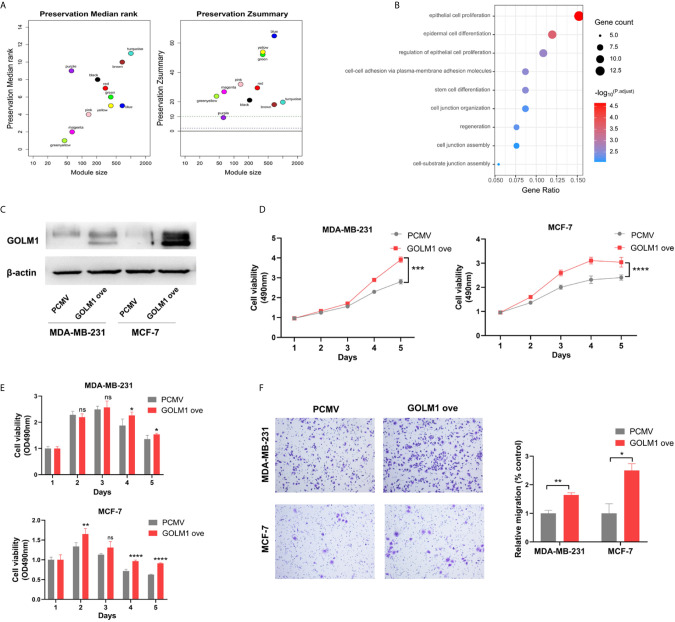
LINC01977 promoted breast cancer progression and chemoresistance to DOX by targeting miR‐212-3p/GOLM1 axis. **(A)** Module preservation analysis in WGCNA. Each module is represented by its color-code and name. Left plot shows the preservation median rank. Right plot shows the preservation Zsummary value. Zsummary value <10 represented lowly preserved modules. **(B)** Gene ontology analysis. **(C)** Expression efficiency of GOLM1 was validated by western blotting assay. **(D)** MTT assay. **(E)** Cytotoxicity assay. **(F)** Cell migration array. (*p < 0.05, **p < 0.01, ***p < 0.001, ****p < 0.0001; Student’s t-test).

After module preservation analysis was performed, purple module (Zsummary =9.2) was defined as the lowly preserved module, which represented the special properties of the group with high GOLM1 expression (**Figure 6A**). We selected top100 kME genes associated with purple module and then performed the GO analysis (**Figure 6B**). In the purple module, several GO terms associated with GOLM1 biological processes were detected, mainly including epithelial cell proliferation, epidermal cell differentiation, cell-cell adhesion *via* plasma-membrane adhesion molecules, stem cell differentiation, and cell junction organization.

To further identify GOLM1’s biological function, vectors for control or GOLM1 overexpression were transfected into MDA‐MB‐231 and MCF‐7 cells. The efficacy of GOLM1 overexpression was validated by western blot assay in two cell lines (**Figure 6C**). Next, cell proliferation, chemoresistance, and migration were evaluated using MTT, cytotoxicity, and transwell assays, respectively (**Figures 6D–F**). Our results demonstrated that GOLM1 could promote cell proliferation, metastasis, and resistance to DOX.

## Discussion

With the development of RNA sequencing, thousands of lncRNAs have been demonstrated to participate in various biological processes, including tumor malignant progression (11). However, breast cancer-related lncRNAs are still underexplored. In this study, integrated bioinformatics analysis was performed to identify LIN01977 as a key lncRNA in malignant progression of breast cancer. Consistent with a previous report in papillary thyroid carcinoma (31), LINC01977 is also remarkably upregulated in breast cancer tissues compared to their adjacent normal tissues. Moreover, GEO and GDSC datasets were employed to show that high LINC01977 expression was significantly associated with resistance to DOX. Survival analysis indicated that high LINC01977 expression predicted shorter survival time in patients with breast cancer. Thus, the biological role of LINC01977 is worth more investigation in the development of breast cancer.

To continue to explore the biological function of LINC01977, we performed *in vitro* experiments using breast cancer cell lines, which further demonstrated that LINC01977 could promote breast cancer cell proliferation, metastasis, and resistance to DOX. Subsequently, cellular fractionation assay was conducted to determine the subcellular localization of LINC01977. Our result indicated that LINC01977 was predominantly located in cytoplasm. Besides, bioinformatics analysis showed that LINC01977 was able to act as a molecular sponge *via* binding to miR-212-3p. Hence, we speculated that LINC01977 may exert its biological function *via* ceRNA mechanism.

miR-212-3p was previously reported as a tumor suppressor in multiple cancer types. Liu H et al. demonstrated that miR-212-3p suppressed tumor cell growth in glioblastoma by miRNA-mediated gene degradation (32). Additionally, Wada R et al. revealed that miR-212 suppressed the development of gastric cancer *via* MECP2 silencing (33). To validate whether LINC01977 could bind to miR-231-3p as a molecular sponge, we performed RIP, real-time PCR, dual-luciferase report assay, and rescue assay. Our findings indicated that miR-231-3p can sponge LINC01977 and reverse the oncogenic phenotype of LINC01977.

To further identify a target gene of LINC01977/miR212-3p axis in breast cancer, bioinformatics analysis was performed to define that GOLM1 was a potential target of miR-212-3p. Real-time PCR was used to validate the relationship among the three players. GOLM1, known as GP73 and GOLPH2, can encode a Golgi associated protein, which is a highly-phosphorylated protein located in the cis and medial-Golgi apparatus (34). Many studies have suggested that GOLM1 can function as a promoter of oncogenic phonotype in several cancer types (35). Ye QH et al. proved that GOLM1 played a key role in cell cycle and metastasis of hepatocellular carcinoma (HCC) cells, and may serve as a prognostic indicator and therapeutic target in HCC patients (36). In our study, WGCNA was carried out to evaluate the biological role of GOLM1 in breast cancer. Purple module in the group with high GOLM1 expression was defined as non-preservation module. Subsequent GO analysis indicated that the biological processes related to GOLM1 were mainly enriched in epithelial cell proliferation, epidermal cell differentiation, cell-cell adhesion *via* plasma-membrane adhesion molecules, stem cell differentiation, and cell junction organization. Next, we further confirmed that GOLM1 overexpression could evidently promoted breast cancer cell proliferation, metastasis, and resistance to DOX by *in vitro* experiments.

Our data presented here suggested that LINC01977 was an attracting therapeutic target for breast cancer treatment. However, this study was mainly based on human breast cancer cell lines, and lack of the xenograft tumor model and other non-clinical test. Addressing this issue will be a critical direction for our future work.

In summary, LINC01977 was identified as a key oncogenic driver in breast cancer, and significantly promoted breast cancer cell proliferation, metastasis, and chemoresistance to DOX. Moreover, our results demonstrated that LINC01977 may exert its biological effect by targeting miR-212-3p/GOLM1 axis, which broaden our insights into the post-transcriptional regulation mechanism and help provide a novel prognostic indicator and therapeutic target for patients with breast cancer.

## Data Availability Statement

Publicly available datasets were analyzed in this study. These can be found in TCGA (http://cancergenome.nih.gov/), GDSC (https://www.cancerrxgene.org/), GTEX (http://xena.ucsc.edu/), and GEO (https://www.ncbi.nlm.nih.gov/geo/) (GPL22755 platform: GSE155478).

## Author Contributions

QY conceived the project. ZL and YL designed the study and performed the experiments. ZL wrote the manuscript and analyzed the data. ZL, YL, and QY reviewed the data and proofread the manuscript. XW, YL, DL, DH, CL, TC, HZ, YL, ZW, BC, LW and WZ assisted the experiments. All authors contributed to the article and approved the submitted version.

## Funding

This work was supported by National Key Research and Development Program (No. 2020YFA0712400), Special Foundation for Taishan Scholars (No. ts20190971), National Natural Science Foundation of China ( No. 81874119; No. 82072912), Special Support Plan for National High Level Talents (Ten Thousand Talents Program W01020103), National Key Research and Development Program (No. 2018YFC0114705), Foundation from Clinical Research Center of Shandong University (No.2020SDUCRCA015), Qilu Hospital Clinical New Technology Developing Foundation (No. 2018-7; No. 2019-3).

## Conflict of Interest

The authors declare that the research was conducted in the absence of any commercial or financial relationships that could be construed as a potential conflict of interest.
