# Supplementary figures and images for: LINC01977 Promotes Breast Cancer Progression and Chemoresistance to Doxorubicin by Targeting miR-212-3p/GOLM1 Axis
Source: Front Oncol. 2021 Mar 31;11:657094. doi: 10.3389/fonc.2021.657094 (PMC8046671; doi:10.3389/fonc.2021.657094)

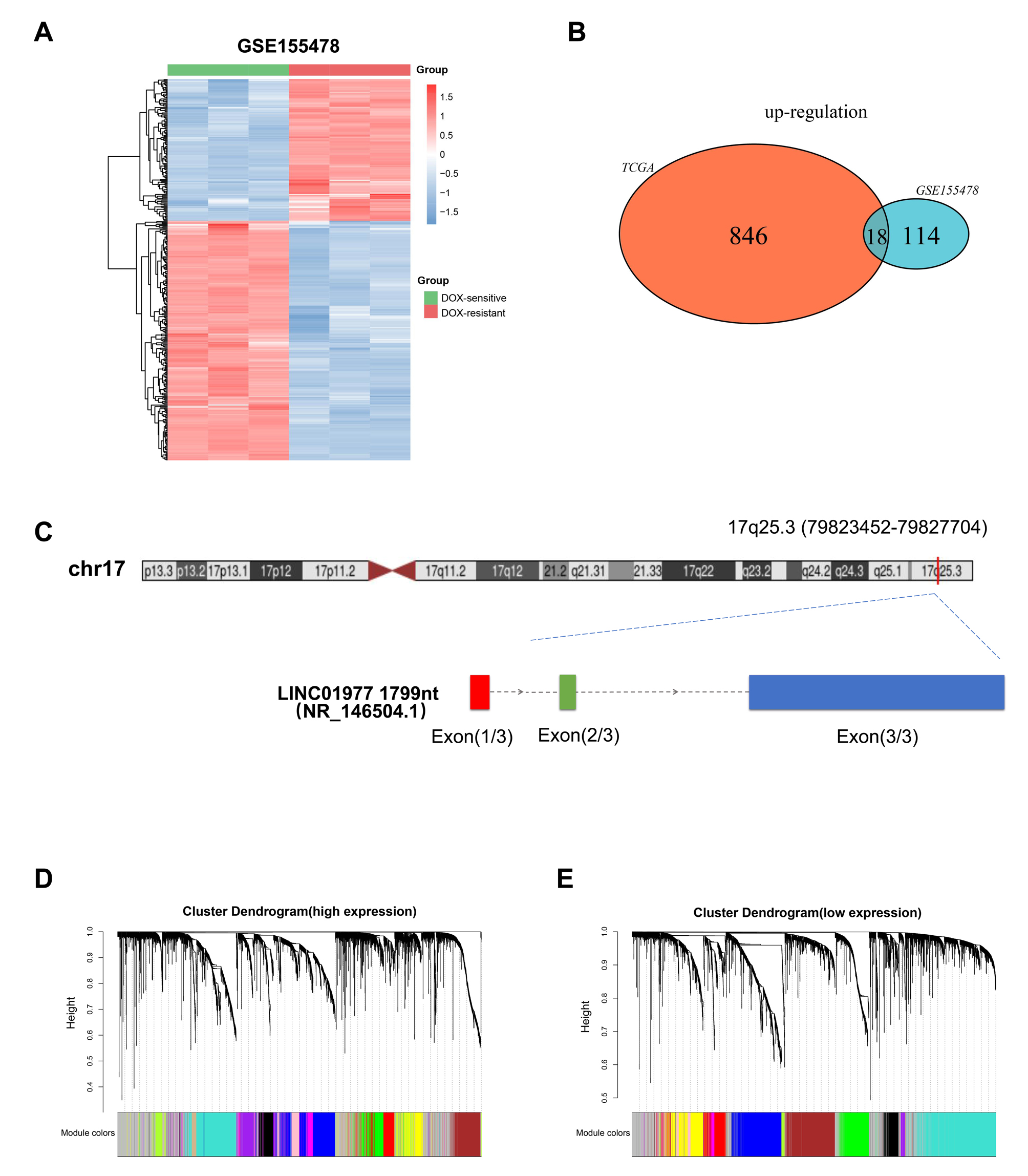

Supplement: Supplementary file 1 [file Image_1.jpeg]
