# Supplementary material for: LINC01977 Promotes Breast Cancer Progression and Chemoresistance to Doxorubicin by Targeting miR-212-3p/GOLM1 Axis
Source: Front Oncol. 2021 Mar 31;11:657094. doi: 10.3389/fonc.2021.657094 (PMC8046671; doi:10.3389/fonc.2021.657094)
Supplement: Supplementary file 2 [file Table_1.docx]

**Supplementary Table 1. Primers used for real-time PCR.**

| **Gene** | **Forward （5’-3’）** | **Reverse （5’-3’）** |
| --- | --- | --- |
| LINC01977 | GGACACTGGTTTACGAAAGT | AATGACACGGCTCTACGC |
| GOLM1 | AAAAGCGGAATCATACAC | TCTCACGAAATACCTACTACC |
| Actin | CATGTACGTTGCTATCCAG | CTCCTTAATGTCACGCACG |
| miR-212-3p | TAACAGTCTCCAGTCACGGCC | CAGTGCGTGTCGTGGAGT |
| U6 | CTCGCTTCGGCAGCACA | AACGCTTCACGAATTTGCGT |
