# Supplementary material for: LINC01977 Promotes Breast Cancer Progression and Chemoresistance to Doxorubicin by Targeting miR-212-3p/GOLM1 Axis
Source: Front Oncol. 2021 Mar 31;11:657094. doi: 10.3389/fonc.2021.657094 (PMC8046671; doi:10.3389/fonc.2021.657094)
Supplement: Supplementary file 3 [file Table_2.docx]

**Supplementary Table 2. Antibodies used in the experiments.**

| **Antigen** | **Supplier** | **Catalog #** | **Application** |
| --- | --- | --- | --- |
| GOLM1 | Proteintech | 15126-1-AP | WB (1:1000) |
| Ago2 | Millipore | 2729982 | WB (1:1000) |
| β-actin | Proteintech | 60008-1-Ig | WB (1:2000) |
| HRP-anti-mouse | CST | 7076 | WB (1:5000) |
| HRP-anti-rabbit | CST | 7074 | WB (1:5000) |
